# An Integrated Data Driven Approach to Drug Repositioning Using Gene-Disease Associations

**DOI:** 10.1371/journal.pone.0155811

**Published:** 2016-05-19

**Authors:** Joseph Mullen, Simon J. Cockell, Peter Woollard, Anil Wipat

**Affiliations:** 1 Interdisciplinary Computing and Complex BioSystems (ICOS) Research Group, School of Computing Science, Newcastle University, Newcastle upon Tyne, United Kingdom; 2 Bioinformatics Support Unit, Newcastle University, Newcastle upon Tyne, United Kingdom; 3 Computational Biology Department, Quantitative Sciences, GlaxoSmithKline Research & Development Ltd, Stevenage, Hertfordshire, United Kingdom; University of Turin, ITALY

## Abstract

Drug development is both increasing in cost whilst decreasing in productivity. There is a general acceptance that the current paradigm of R&D needs to change. One alternative approach is drug repositioning. With target-based approaches utilised heavily in the field of drug discovery, it becomes increasingly necessary to have a systematic method to rank gene-disease associations. Although methods already exist to collect, integrate and score these associations, they are often not a reliable reflection of expert knowledge. Furthermore, the amount of data available in all areas covered by bioinformatics is increasing dramatically year on year. It thus makes sense to move away from more generalised hypothesis driven approaches to research to one that allows data to generate their own hypothesis. We introduce an integrated, data driven approach to drug repositioning. We first apply a Bayesian statistics approach to rank 309,885 gene-disease associations using existing knowledge. Ranked associations are then integrated with other biological data to produce a semantically-rich drug discovery network. Using this network, we show how our approach identifies diseases of the central nervous system (CNS) to be an area of interest. CNS disorders are identified due to the low numbers of such disorders that currently have marketed treatments, in comparison to other therapeutic areas. We then systematically mine our network for semantic subgraphs that allow us to infer drug-disease relations that are not captured in the network. We identify and rank 275,934 drug-disease has_indication associations after filtering those that are more likely to be side effects, whilst commenting on the top ranked associations in more detail. The dataset has been created in Neo4j and is available for download at https://bitbucket.org/ncl-intbio/genediseaserepositioning along with a Java implementation of the searching algorithm.

## Introduction

Understanding the molecular mechanisms of diseases is vital within the field of target-based drug discovery. A causal association between a gene and a disease describes a situation whereby a gene is directly or indirectly responsible for disease risk via one or more mechanisms [1]. Monogenic disorders, such as Huntington’s disease, are identified simply through the presence, or absence, of single gene mutations; in this case a mutation in the Huntingtin protein, HTT [2]. Conversely, multigenic, or complex, disorders are caused by multiple genetic variants, which may affect pleiotropic genes and be influenced by various environmental factors [3]. Due to the complexity of multigenic diseases, allele associations are more probabilistic and less deterministic; the presence of a high-risk allele may only mildly increase the chance of disease [3][4]. For these reasons identifying causal links between a gene and disease experimentally is expensive and time consuming. Association studies, however, identify disease susceptibility variants that do not necessarily mean the variant is important in disease causation. It is an easier task to identify gene-disease (G-D) associations as opposed to causation associations [5].

The shift to large scale sequencing of individual genomes and the availability of new techniques for probing thousands of genes provide new means for identifying these G-D associations. Experimental techniques such as positional cloning and/or microarray analysis can return tens to hundreds of candidate genes [6]. Managing and integrating these data has thus become an important task within bioinformatics, and numerous G-D databases have been developed to aid this. Entries in databases are mainly obtained through manual curation of the biomedical literature [7]. In order to capture data that may have been missed by manual curation, automated text mining approaches can also be used [8]. Although automated text mining approaches improve recall, precision is drastically reduced in comparison to manual extraction. Genetic associations can also be extracted directly from experimental data, such as genome-wide association studies (GWAS), and stored in dedicated databases. Predictive methods may also be used to populate databases identifying associations through statistical inference, including cross species inferences derived from animal models. Mouse and rat models have been used to predict human G-D associations for a number of years, and there exists a wealth of cross-species G-D association data available [9–11]. Cross-species models can be complicated by diverse types of phenotype representations in terms of physiological and anatomical differences between species, however this knowledge cannot be ignored [12]. In order to create a state of the art view of current knowledge regarding G-D associations, integration of these heterogeneous data-sources is required.

A holistic view of the field allows for emergent properties that would otherwise be invisible to be realised [13]. Secondary resources, such as DisGeNET [14, 15] and MalaCards [16], already integrate associations from multiple primary resources that have been curated, predicted and derived computationally from text. DisGeNET apply a systematic scoring to these associations, however the chosen metric fails to give a relative view of known G-D associations. A complete ranking of G-D associations from primary resources, taking into consideration the reliability of each dataset using current knowledge, would aid tasks such as computational target-based drug discovery [17], as well as reducing inevitable bias present in datasets that were all developed for different purposes. Despite historically being driven by phenotypic approaches [18], target-based approaches to drug discovery came to prominence after sequencing of the human genome. It was believed that target-based drug discovery would allow for a more rational approach to drug design, and thus increase research and development (R&D) success and productivity [18, 19]. Target-based approaches are still heavily prominent and extensively used in the pharmaceutical industry [20], with successes including the tyrosine kinase inhibitors imatinib (Gleevec) and gefitinib (Iressa) [21]. Overall, due to increased costs and reduced productivity, there is a general acceptance that the current state of R&D needs to change [22].

Part of the solution, in the short term, is drug repositioning, also known as drug repurposing. Drug repositioning is the application of established, approved compounds to treat diseases other than those for which they were marketed. This process allows for increased confidence and reduced attrition further along the development pipeline, resulting in reduced development costs and time taken for a drug to reach the market. Many repositioned drugs currently on the market have been discovered through serendipitous or rational observations, as demonstrated by sildenafil (Viagra) and duloxetine (Cymbalta) respectively. Neither of these drugs utilised efficient routes to market given the potentially huge search space of drug-disease interactions. Systematic approaches to the searching of such solution spaces are required to provide an efficient and scalable alternative to manual investigations. As a means of satisfying these needs many pharmaceutical companies now have groups focused purely on repositioning. Academic interest has also resulted in numerous studies describing systematic computational approaches to drug repositioning. Existing methodologies are based on: chemical structure similarity [23]; protein structure similarity and molecular docking [24]; phenotype similarity (including side-effect similarity [25] or gene expression similarity [26]); and genetic variation [27]. Numerous network-based approaches focus on the creation or mining of integrated networks that allow for many of these approaches to be implemented or even combined [28, 29]. Data integration is an essential part of systems analysis; providing integrative views of multiple data sources and data types, such as drugs, proteins, genes and diseases [30].

Chiang *et al* [31], for example, integrate data describing diseases and drugs. A network-based guilt-by-association (GBA) method is also introduced, whereby novel drug uses are inferred based on a shared treatment profile of disease pairs. This approach takes a very high-level view of the field, focussing purely on drug-disease relations with no consideration of the underlying genetic or pharmacological mechanisms at play. Gottlieb *et al* [32] make use of a broader collection of datasources to create five drug-drug similarity measures and two disease-disease similarity measures. These similarity measures are then used by PREDICT, an algorithm to infer novel drug indications. Other approaches utilise target information during the prediction task and the associations between these and the disease state. Huang *et al* [33] integrate drug, protein and disease data. A network propagation model is then used to infer potential drug-protein/G-D relationships, in which genes with similar functional modules are related to drugs. Unlike the other approaches, Daminelli *et al* [34] introduce a method that focusses on known data to ‘fill in the blanks’, as opposed to using abstracted similarity data. Recognising the importance of G-D associations this approach integrates structural and chemical data to build a drug-target-disease network. This network is then mined for network motifs of bi-cliques, where every drug is linked to every target and disease. Links from drugs to diseases are predicted by completing the incomplete bi-cliques. Interestingly, the authors chose to focus the approach on only 147 promiscuous drugs.

In this work we introduce an exhaustive, novel approach for identifying new uses for existing drugs, with a focus on G-D associations. We apply a Bayesian statistics approach, developed by Lee and colleagues [35], as means of integrating and ranking G-D associations captured in 10 primary data sources. Scored G-D associations, providing a state of the art view of G-D knowledge, are then integrated with other biological entities to produce a semantic network for target-driven drug repositioning. A method for the automated detection of therapeutic areas of interest is also introduced. Finally we introduce a four node semantic subgraph and mine the integrated network for instances of this subgraph, using an algorithm previously described by Mullen *et al* [36]. Novel drug-disease interactions inferred from the network are then ranked, with those involving diseases from the therapeutic area of interest discussed in more detail. It is expected that the approach introduced in this paper will facilitate further research on drug repositioning.

## Background

### Gene-disease association databases

Several existing primary databases focus on G-D associations. These databases typically contain associations obtained through manual curation of the biomedical literature. One well established source of G-D associations is the Online Mendelian inheritance in Man (OMIM) database [37]. More recent projects include the Comparative Toxigenomics Database (CTD) [38] and UniProtKB [39]. Another source, Orphanet [40], focusses primarily on rare diseases and orphan drugs. Databases populated with associations extracted directly from the literature, using text mining approaches, also exist [7], such as BeFree [8] and SemRep [41]. Although the accuracy of automatically extracted associations is not as high as manually curated data, the systematic approach to their construction means that they are more inclusive of true positives.

BeFree [8] provides a good example of a text mining resource. BeFree, along with supporting statements and provenance, is available for download and uses the EU-ADT and GAD corpora to extract associations from text. Focussing on a subset of abstracts returned from PubMed, BeFree use their own query (only querying about 3% of current MEDLINE databases). After applying filtering, BeFree captures 330,888 associations involving 13,402 genes and 10,557 diseases [8]. SemRep [41] also provides text mined associations. Like BeFree, SemRep provides gd, drug-disease and drug-target associations, but unlike BeFree has been designed to identify a large variety of semantic predictions. When using the same corpus as BeFree, SemRep has a higher precision but a lower recall [8]. Other approaches to collecting G-D associations involve cataloguing data directly from genetic experiments, or inferring associations from animal models.

Over the last decade GWAS have produced data on thousands of single nucleotide polymorphisms (SNPs). These SNPs are associated with the risk of hundreds of diseases. Although developed as a means to identify causal SNPs, GWAS data are non-trivial to work with; they identify marker SNPs that are often not the causal, rather associative, and present as a consequence of the disease state as opposed to being responsible for causing the disease state. It is also worth noting that GWAS data only contains associations derived from a subset of diseases for which genetic studies have been conducted. As with any exercise in data collection, the data captured in datasources may be biased, depending on the intended purpose of the data. This is particularly true of GWAS data, which is particularly biased [42] to diseases such as Crohn’s disease that are of interest to industry. Nevertheless, GWAS data are available for download via the GWAS catalogue [43]. The Rat Genomics Database (RGD) [10] and the Mouse Genomics Database (MGD) [9] provide G-D associations that have been identified in animal models but are statistically inferred to represent human associations.

### Controlled vocabulary of diseases

Before dealing with G-D associations it is important to identify a standardised representation of both genes and diseases. Due to work completed by the Human Genome Organization (HUGO) Gene Nomenclature Committee (HGNC), it is a fairly straightforward task to identify a strict representation for human genes [44]. To identify a good disease representation is more complicated, since there are numerous disease classifications and ontologies competing with one another. These disease classifications are designed for different purposes and are mutually inconsistent, consequently these are poorly integrated with each other. The Systematized Nomenclature of Medicine-Clinical Terms (SNOMED-CT) is one such example and cross maps to several revisions of the International Classification of Diseases, which is used in the clinical setting [17]. SNOMED-CT is one of the many terminologies that is combined in the even broader Unified Medical Language System (UMLS) Metathesaurus; another is Medical Subject Headings (MeSH) [45]. UMLS contains many distinct concepts that are very close in meaning, and as a result even human annotation using UMLS concepts is problematic [46].

One alternative is the Disease Ontology (DO), part of the Open Biomedical Ontologies (OBO) Foundry Initiative. The DO cross maps to UMLS and has extensive cross-referencing, however it maps poorly to diseases captured in datasets such as DisGeNET. We recently calculated that of the diseases captured in DisGeNET, 100% mapped to UMLS, 60% mapped to MeSH and 24% mapped to the DO. This is a current challenge for large-scale disease data integration that aims to gather a comprehensive coverage of disease to enable systematic interoperability across biomedical domains [8]. At present, it appears that MeSH offers the best trade off between interoperability and semantic clarity.

### Graph Model

In order to view G-D associations in biological context it is important to define a data structure that will aid in this task. Graph representations of complex systems are widely used in computer science, social and technological network analysis, and are particularly relevant to many studies in bioinformatics [47]. Semantically-rich networks, which implement a graph-based representation, are ideal for representing integrated data [48]. In *semantic graphs* each edge (or relation) and vertex (or node) are assigned a single type from a predefined set to semantically describe their meaning. In such a representation, vertex v_1_ may represent cGMP-specific 3’,5’-cyclic phosphodiesterase and is assigned the type Protein, whilst vertex v_2_ represents sildenafil and is thus assigned the type Small_Molecule. If v_1_ is a known target of v_2_ we capture this interaction in a directed edge, e_1_, of type binds_to. Vertices and edges of semantic graphs may also be annotated with attributes.

## Materials and Methods

**Fig 1:**
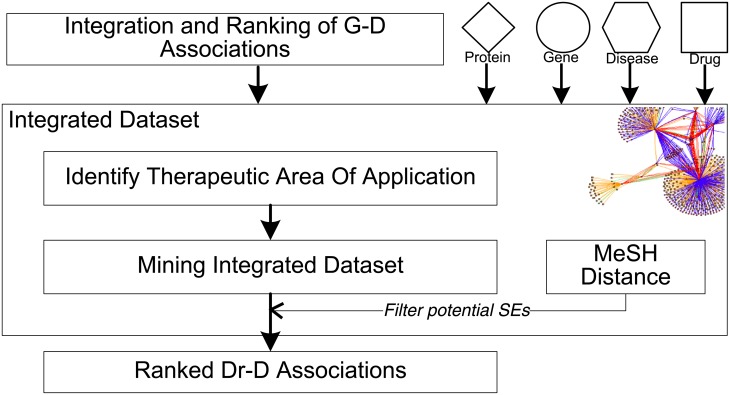
Overview of approach to identify novel drug-disease (Dr-D) associations. Gene-disease associations from 10 sources are first integrated and ranked. These scored associations are then integrated with protein, gene, disease and drug data to give an integrated dataset. A therapeutic area of application is then identified before the dataset is mined for instances of a semantic subgraph whose mappings contain inferred dd associations. Finally, any dd associations that are likely side effects (SEs) are filtered using the MeSH distance measure, before all ranked dd associations are returned.

We have developed an approach to identify novel drug-disease (Dr-D) associations from an integrated target network, showing how data-inspired hypothesis generation can be used to guide mining. The approach is made up of five main components which are described in Fig 1. These components comprise: (i) Integration and ranking of G-D associations (ii) The creation of a semantic integrated network for target-based drug repositioning, using scored G-D associations, protein, gene, disease, and drug data (iii) Identifying a therapeutic area of application, using only the integrated network (iv) Mining the integrated dataset for instances of a semantic subgraph whose mappings allow us to infer novel uses for existing drugs (v) A method for calculating semantic distance between two diseases within the MeSH hierarchy.

### Integrating Gene-Disease Associations

**Fig 2:**
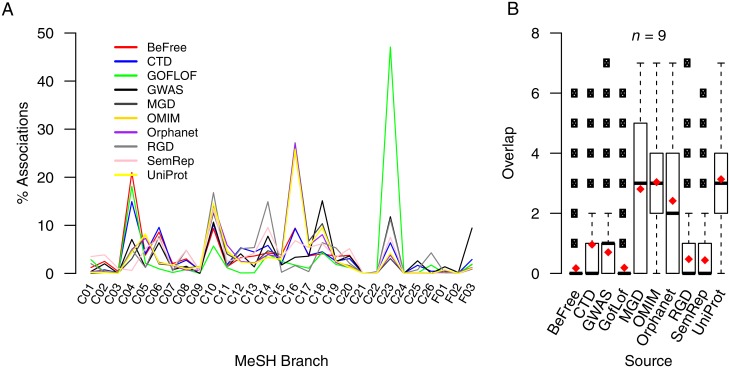
Comparison of gene-disease (G-D) sources. (A) shows the percentage spread of G-D associations from each integrated datasource across the 29 MeSH disease branches. (B) a boxplot showing the overlap of G-D associations between the ten datasources. *x* associations were picked at random and overlap between the other data sources identified (*x* = 1000). *Note*: *n* = number of data sources checked, red diamonds show the mean, open circles are outliers and the median is represented by the thick horizontal black lines.

In order to avoid data duplication and redundancy we focus only on primary data resources and do not include secondary resources, such as DisGeNET and Malacards during G-D association integration. In an effort to reduce bias in the data we include data from sources that cover all four of the database types described previously: curated, experimentally derived, literature derived, and those inferred from animal models. G-D associations were extracted from the sources listed in Table 1. Only G-D associations that contained diseases mappable to the MeSH hierarchy were included in the analysis. Mapping between UMLS^®^ Concept Unique Identifiers (CUIs) and MeSH was done using the Metathesaurus^®^. This mapping was used for associations captured in BeFree, CTD and SemRep. Next, all 2,208 mappings present between MIM and MeSH identifiers were extracted from ORDO. This set of mappings was extended to 3,967 using a manually curated mappings set of 3,029 (with overlap). This mapping between MIM and MeSH was then used to integrate associations captured in MGD, OMIM and UniProtKB. For G-D associations in GWAS a manually curated mapping between 1,131 GWAS traits and MeSH Unique Identifiers (UIs) was used (see S1 Data). G-D associations from RGD required no mapping as diseases are already categorised using MeSH UIs. After all G-D associations were mapped to MeSH, a relatively even spread of G-D associations across all 29 therapeutic areas of the MeSH hierarchy (see S1 Table for MeSH category names) was observed, with C04, C10, C16 and C23 being slightly over represented (Fig 2A). We can also see that associations from OMIM and UniProtKB are, on average, captured in more than three of the other datasources, whilst, on average, there is little crossover between associations captured in BeFree, GoF/LoF, RGD and SemRep (Fig 2B).

**Table 1:** Data sources of gene-disease associations.

Source	Version/Accessed	Type	#Associations	#Map MeSH	% Map MeSH
CTD [50]	Jul_02_2015/Aug’15	Curated	24,346	23,813	97.8
OMIM^®^ [37]	18-08-2015/Aug’15	Curated	5,143⋆	3,375	65.6
Orphanet [40]	2015_07_31/Jul’15	Curated	6,094	1,744	28.6
UniProtKB [39]	2015_08	Curated	4,679	3,203	68.5
GWAS Catologue [43]◇	24_08_2015/Aug’15	Experimental	13,326	5,112	38.4
BeFree [8]	24-Aug-2015/Aug’15	Literature	330,888	233,264	70.5
GoF/LoF⊲	-/Oct’15	Literature	4,793	3,459	72.2
SemRep [41]∘	25/Feb’15	Literature	96,024	72,908	75.9
MGD [9, 11]*	24_08_2015/Aug’15	Predicted	1,943	1,577	81.2
RGD [10]*	21_08_2015/Aug’15	Predicted	7,667	7,667	100

Datasources used for G-D associations. ‘Curated’ refers to manually curated associations, ‘Experimental’ refers to associations drawn directly from genetic experimental observations, ‘Literature’ refers to associations automatically mined from literature and ‘Predicted’ refers to associations statistically inferred from animal models.

^⋆^ Not including 1,397 associations for which the molecule basis is unknown.

^◇^Threshold of 1e-7 was used.

^⊲^See S1 Article.

^∘^Extracted associations between gene and disease that were of the following predicates: AFFECTS; ASSOCIATED_WITH; AUGMENTS; CAUSES; PREDISPOSES; COEXISTS_WITH and NEG_ASSOCIATED_WITH as described in [8].

*Used the same parameters used by DisGeNET to extract predicted associations.

### Ranking Gene-Disease Associations

Gold standards are used as a reference point for many predictive and scoring methodologies, and are generally a set of consensus knowns that have been agreed upon by the community. For areas whereby a gold standard does not exist, such as the G-D setting, this set becomes subjective to the area of use and the task at hand. Different approaches exist for ranking disparate data; some do not use a a gold standard, such as that described by Weile and colleagues [49], and some that do make use a gold standard, like the work completed by Lee and co-workers [35]. In order to score individual G-D associations we used the Bayesian statistics approach that makes use of a gold standard, developed by Lee and colleagues [35]. This approach calculates a log-likelihood score (LLS) for each dataset, using Eq 1:

$$\begin{array}{c} LLS^{L}\left(E\right)=log\left(\frac{P(L|E)/\neg P(L|E)}{P(L)/\neg P(L)}\right) \end{array}$$ (1)

where *P*(*L*|*E*) and ¬*P*(*L*|*E*) are the frequencies of edges, or links, (*L*) observed in a given datasource (*E*) between genes and diseases. For estimating these conditional odds, we count the number of G-D pairs that have associations and are also supported by the gold standard. This score can therefore be interpreted as the likelihood of the linkage conditioned on the given evidence and corrected for background expectations of linkages. In Bayesian terms, the ratios *P*(*L*)/¬*P*(*L*) represents the *prior* odds ratio, which is the ratio of the probability of the linkage and its negation before the evidence is seen. The log likelihood score can be interpreted as being proportional to the accuracy of the datasource. This term is estimated by counting the number of G-D pairs with a known interaction and those without any shared annotation among all possible G-D pairs captured in the data.

The confidence scores were then integrated using the weighted sum (WS) as described by [35] and summarised in Eq 2. In Eq 2, C_1_ is the highest confidence score and C_n_ the lowest confidence score computed from a set of n datasets. A higher weighting is given to datasets with higher confidence, which facilitates dependencies between the datasets. Division of the score by a computed D parameter means that, while the highest score is integrated unchanged, subsequent LLS scores are progressively down-weighted. This is especially relevant to G-D associations, whereby it is common practice to primarily populate a database with associations from other curated sources before extending it (CTD, Orphanet and UniProtKB all collect a subset of associations captured in OMIM).

$$\begin{array}{c} WS=\sum_{i=1}^{n}\frac{C_{i}}{\left(D^{i-1}\right)} \end{array}$$ (2)

### Integrated Dataset

Ranked and scored G-D associations were then integrated with other data to create a semantically-rich network to aid in the identification of potential drug repositioning opportunities (all sources and data types included in the network are detailed in Table 2). The dataset was built using the Neo4j Java API version 2.1.2 and, after removing all unconnected entities, contains 55,973 nodes and 529,738 edges (a TSV version of the dataset is provided S2 Data). To distinguish between rare (generally monogenic) and common (often complex) diseases the Orphanet Rare Disease Ontology (ORDO) was also included. 1,779 MeSH UIs were captured as synonyms within the ORDO. Wherever a Rare_Disease node contained a MeSH UI, the MeSH node was integrated with the ORDO disease and resulted in a Rare_Disease vertex with the synonymous MeSH UI becoming an attribute. The metagraph for our dataset is shown in S1 Fig.

**Table 2:** Data sources, types, attributes and frequency used in integrated repositioning graph.

Source	Version/Acc	NodeType	#Nodes	RelationType	#Rels	Attributes
UniProtKB [39]	2015_08	Protein	20,203	-	-	UniProt UID
						UniProt ID
						Name
UniProtKB	2015_08	Gene	19,744	-	-	Entrez Gene Symbol
						Entrez Gene ID
UniProtKB	2015_08	-	-	encoded_by	19,903	-
ORDO [51]	2/July’15	Rare_Disease	8,626	-	-	Name
						MESH
						OMIM
						UMLS
ORDO	2/July’15	-	-	part_of	12,518	-
ORDO	2/July’15	-	-	has_parent	11,201	-
MeSH [45]	2015/Aug’15	Common_Disease	11,735*	-	-	MeSH Header
						MeSH
						MeSH Tree
MeSH	2015/Aug’15	-	-	is_a	23,829	-
DrugBank [52]	4.3/July’15	Small_Molecule	7,469	-	-	DBID
						Name
						Category
						Group
DrugBank	4.3/July’15	-	-	binds_to	14,250	Action
ChEMBL [53]	20/Sep’15	-	-	binds_to	23,507	Activity type
						Activity value
ChEMBL	20/Sep’15	-	-	-	-	Drug mechanism⋆
SIDER [54]	4/Aug’15	-	-	has_indication	4,488⊲	-
NDFRT [55, 56]	Aug’15	-	-	has_indication	4,396	-
PREDICT [57]	-	-	-	has_indication	1,265	-
CTD curated [58, 59]	-	-	-	has_indication	18,540	-
SIDER	4/Aug’15	-	-	has_side_effect	67,934∘	-
Scored gd	-	-	-	involved_in	309,885	Association score Directionality◇

Data sources used in the creation of the repositioning dataset.

*Made up of 5,370 descriptor records and 6,365 supplementary records.

^⋆^532 drug activity types (including agonist and antagonist) were taken from ChEMBL and mapped to drugs in the dataset.

^⊲^Unique associations from the 16,306 integrated.

^∘^Unique associations from the 163,525 integrated.

^◇^3,459 G-D associations are annotated with the gene functionality resulting in a disease state, either loss-of-function (2,211) or gain-of-function (1,248).

The integrated graph contains approved drugs, Small_Molecules, and binds_to interactions from these to single Protein targets. Wherever possible these binds_to associations are annotated with activity types (IC_50_, K_d_, K_i_ and Potency) and the corresponding activity values (nM). For each Protein, the Gene which it is encoded_by is also included. A Gene may also be linked to diseases, either a Rare_Disease or a Common_Disease, via involved_in associations. These involved_in associations are annotated with values produced during the G-D association ranking described previously. Finally, diseases and drugs may share has_indication and has_side_effect edges. The dataset is designed in a manner to allow for target-based drug repositioning opportunities to be identified systematically.

### Identifying Area of Application

Using both G-D associations and Dr-D associations from the integrated network we calculated a therapeutic area unmet score (TAU), using the formula in Eq 3;

$$\begin{array}{c} TAU\left(ta\right)=\neg P\left(Dr-D\right)\times P\left(G-D\right)\times \left(1-\frac{1}{MAX}\times \left|ta\right|\right) \end{array}$$ (3)

Where ta is the therapeutic area being looked at (e.g. C01), *P(G-D)* is the probability that the data contains a G-D association for a disease in that ta, *¬ P(Dr-D)* is the probability that the data does not contain a dd association for a disease in that ta, and MAX represents the size of the greatest ta. The TAU, in theory, can range from 0 (therapeutic areas with little knowledge in the dataset, highly drugged areas and areas with few diseases) to 1 (therapeutic areas with relatively high levels of knowledge captured in the data containing a low percentage of diseases with marketed small therapeutic molecules and areas with high numbers of diseases).

We also use a simple equation to calculate a rich therapeutic area (RTA) score. This equation uses the same notation as Eq 3 and can also produce scores from 0 (areas with little knowledge captured in the dataset) to 1 (areas with a lot of knowledge captured in the dataset), using the following:

$$\begin{array}{c} RTA(ta)=P(Dr-D)\times P(G-D) \end{array}$$ (4)

### Calculating MeSH Distance

The MeSH hierarchy is rather verbose, and thus the specificity of terms is a potential problem. Due to the size of the MeSH hierarchy, two diseases may be synonymous yet captured in multiple parts of the taxonomy. Ontology based similarity measures may be structure based (e.g. path length, depth of concept or lowest common subsumer) or content based (whereby you use a corpus of terms and look at information content). We created a semantic distance measure, Sim, using the structure based approach described by Leacock and Chodorow [60] to measure the distance between two diseases in the hierarchy (Eq 5). Although originally developed to measure the distance between nouns in WordNet, an electronic lexical database [60], the method has previously been applied to MeSH [61].

$$\begin{array}{c} Sim\left(C_{i},C_{j}\right)=\left(\frac{1}{MAX}\right)\times \left(-log\frac{Dist(C_{i},C_{j})}{2depth}\right) \end{array}$$ (5)

Where MAX is the maximum mapping score, depth is the max depth of the hierarchy and Dist is the shortest path length between the two concepts, C_i_ and C_j_. Reducing the stringency at which diseases are mapped to others in the MeSH hierarchy allows us to better filter potential noise caused by has_side_effect associations. For example, an inferred has_indication association is made between drugX and diseaseY, whilst a known side effect of drugX is diseaseZ, a child term of diseaseY in the MeSH hierarchy. As one of drugX’s known side effects is semantically similar to the inferred indication, it is fair to assume that drugX is not a reasonable candidate for the treatment of diseaseY. In this instance *Sim*(diseaseY,diseaseZ) would give us a value of 0.768. Using 0.768 as the equivalence threshold (ET) during filtering means all inferred associations that are one node away in the MeSH hierarchy, from known side effects will be removed. Therefore, the Sim value allows for the identification of semantic ‘equivalence’ using a certain threshold or leniency, the ET.

### Mining

**Fig 3:**
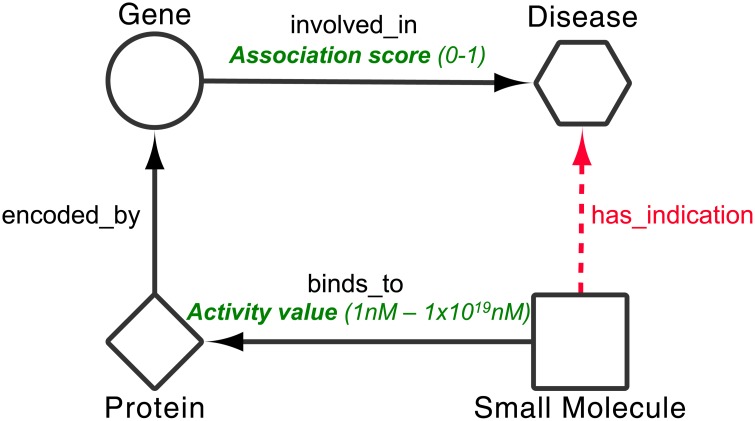
Semantic subgraph used during mining of the integrated network. Subgraph represents the simplest approach to schematically represent the route from drug to disease using target-based approaches to drug repositioning. Through identifying mappings of the subgraph in our integrated dataset we aim to infer the red has_indication relations. Mappings are scored using the values captured in the *Activity value* and *Association score* attributes (shown in green) found on the binds_to and the involved_in relations, respectively. *Note*: in mappings ‘Disease’ can be either a Common_Disease or a Rare_Disease and a ‘Drug’ is an approved Small_Molecule.

An implementation of the semantic subgraph searching algorithm described by Mullen *et al* [36] was used to identify all instances of the semantic subgraph depicted in Fig 3 contained within the integrated network. The algorithm was extended to allow for attribute comparison. The subgraph depicted in Fig 3 was used as it is the most simple schematic representation of a drug-disease pathway. By searching for instances of the four node subgraph we hope to identify novel dd associations, essentially by ‘filling in the blanks’.

Mappings, M, were scored and ranked using the *Activity value* and *Association score* values attached to the involved_in and binds_to relations respectively, using Eq 6. The *Association score* captured on the involved_in relations was created during the G-D ranking section of the approach, and ranged between 0-1. The *Activity value*, attached to the binds_to relation was extracted from ChEMBL and included values associated to: IC_50_; K_i_; K_d_ and potency, all of which had values ranging from 1nM—1 × 10^19^ nM. The *Activity value* for each binds_to association was normalised to the same range as the *Association score*, to give ⋄*Activity value*; this was done simply by subtracting log_10_ (*Activity value*) × 0.1 from 1, where 1 is the maximum log_10_ (*Activity value*) captured in the dataset. At this point is also worth noting that, because no activity values are available in DrugBank, all binds_to relations taken from here are automatically assigned an *Activity value* of 0.8. This value is assigned so as to not miss any potential mappings that are made up of binds_to associations from DrugBank, whilst not over weighting the unknown activity values.

$$\begin{array}{c} Score\left(M\right)=\frac{⋄Activity\;value\;(M)+Association\;score\;(M)}{2} \end{array}$$ (6)

## Results

### Identifying Area of Application

**Fig 4:**
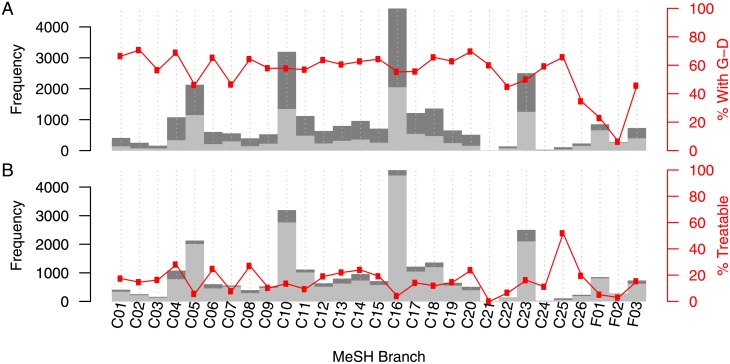
Identifying a therapeutic area of interest. (A) Dark grey shows the number of diseases in each therapeutic area of the MeSH hierarchy. Light grey shows the number of those diseases that are not involved in any of the gene-disease associations captured in our network. Red shows the number of diseases that are involved in a gene-disease association as a percentage of the total number of diseases in that therapeutic area. (B) Dark grey shows the number of diseases in each therapeutic area of the MeSH hierarchy. Light grey shows the number of those diseases that currently do not have a small molecule treatment on the market. Red shows the number of diseases that do have a treatment on the market as a percentage of the total number of diseases in that therapeutic area. *Note*: please see S1 Table for disease area names.

We wished to identify a therapeutic area of unmet need to apply our approach in a data objective manner. In order to do this we looked at two relevant data types from our network, G-D associations and dd associations. As our approach utilises G-D associations for target-based drug repositioning, it is important to target therapeutic areas for which a large proportion of the contained diseases have data supporting their genotypic mechanisms; we cannot infer indications involving therapeutic areas that have no network date. The percentage of each therapeutic area that was involved in at least one involved_in association was therefore calculated; this is shown in Fig 4A. It was then necessary to identify a therapeutic area that had G-D associations describing the diseases, but also had fewer small therapeutic molecules. We therefore calculated the percentage of each area for which there already exists a marketed small therapeutic molecule. This percentage was calculated using the has_indication relations present in our network and is shown in Fig 4B).

When calculating a therapeutic area which has a relatively large amount of knowledge captured in our dataset, we have to also consider the size of said therapeutic area (i.e. how many diseases are contained). For example, the therapeutic area C25 (chemically-induced disorders) has dd associations for 52% of the disorders which are contained within the term, as well as G-D associations for 65%. Looking at these two values alone we can see that there is a relatively large amount of data for this area, however, it is made up of only 108 diseases, and makes up only 0.4% of diseases in the dataset. To avoid identifying such small areas for focus we only consider therapeutic areas that represent over 3.44% (we have 29 therapeutic areas and so 100/29) of the total diseases captured in the dataset to identify a rich therapeutic area.

With a TAU (Eq 3) of 0.53, we show that C16 (hereditary diseases) is the largest unmet therapeutic area (S2 Fig). However, we chose C10 (diseases of the central nervous system), with second highest TAU of 0.38, for the focus of this work. Our work focusses on approved small molecules, as these drugs have already passed safety tests and are easier to reposition. Many genetically simple hereditary diseases are generally not suited to this type of treatment, as some are untreatable and others are caused by gene knock outs. Hereditary diseases tend to be better treated using metabolic manipulation, protein augmentation and gene therapy [62]. We also identify the therapeutic area C04 (Neoplasms) as having the greatest RTA (Eq 4) of therapeutic areas containing more than 3.44% of the total number of diseases in the dataset (S3 Fig). We therefore apply our approach to C10, an area of unmet need, and C04, an area relatively rich in data.

### Ranking Gene-Disease Associations

When using each dataset as the gold standard UniProt, on average, ranked first for the score attributed by the LLS (S2 Table). Because of its consistent high ranking, G-D associations from UniProt were used as the gold standard for the scoring of associations. Using UniProt, LLS scores for the datasets ranged from 16.57 for OMIM to 10.95 for GWAS. After testing a range of D parameters, a D value of 5.0 was used for this work as it was deemed to optimise the area under the curve (AUC) value (S4 Fig). This resulted in a total of 309,885 unique scored G-D associations (S3 Data), with scores ranging from 10.95 to 20.29.

### Mining

The dataset takes 64 minutes to build on on a local machine (8GB RAM and 1.8GHz Intel Core i5). The algorithm described in [36] was implemented in Java and ran against the Neo4j graph. This search used an initial candidate set of 1,188 nodes (approved, small molecule drugs that target humans or other mammals) and took 13 minutes to complete. An exhaustive search returned 539,162 mappings.

Steps were then taken to filter these results in order to remove as much noise as possible. Mappings containing predicted has_indication associations that were known to be side effects (captured as has_side_effect relations in the network) were removed. We also dismissed mappings that predicted has_indication associations with a Sim value ≥ 0.768 to known has_side_effect associations as being potential side effects. An equivalence threshold of 0.768 was used as it gave us the best balance between precision and recall of the known has_indication associations whilst also pruning, on average, the highest ranked inferred associations (S3 Table and S5 Fig). Of the 539,162 mappings, 42,689 were classed as being potential side effects. A further 4,947 mappings were removed as the mechanism of the drug and the G-D association directionality (loss-of-function (LoF) or gain-of-function (GoF) data) contradicted one another (e.g. the drug was an antagonist and gene is associated to disease via a LoF relation). Finally, 41,798 mappings containing one of the 298 absorption, distribution, metabolism, and excretion (ADME) genes [63] were also dismissed.

This left us with 451,269 mappings inferring potential has_indication associations. This set of mappings identified 275,934 unique associations (some associations were identified by more than one mapping) and are provided in S4 Data. Of all the mappings that inferred the same has_indication association, the mapping that achieved the highest score was kept and used for all analysis. Inferred indications covered every therapeutic area of the MeSH hierarchy, ranging from 55,875 for neoplasms (C04) to 2 for disorders of environmental origin (C21) (S1 Table). 219,623 unique associations involved Common_Disease (inferred from 369,124 mappings) whilst 56,311 associations involved Rare_Diseases (inferred from 82,145 mappings) (see Table 3).

**Table 3:** Number of mappings for each disease type and therapeutic area post filtering.

	All Diseases	Common Disease	Rare Disease
**All Therapeutic Areas**	275,934 (451,269)	219,623 (369,124)	56,311 (82,145)
**C04: Neoplasms**	55,875 (102,832)	39,383 (73,501)	16,492 (29,331)
**C10: Nervous System Diseases**	54,635 (84,213)	41,241 (66,536)	13,394 (17,677)

After applying filtering we were left with a set of mappings that inferred unique (no repeats) drug-disease associations. Numbers in brackets denote how many mappings inferred the unique associations.

**Fig 5:**
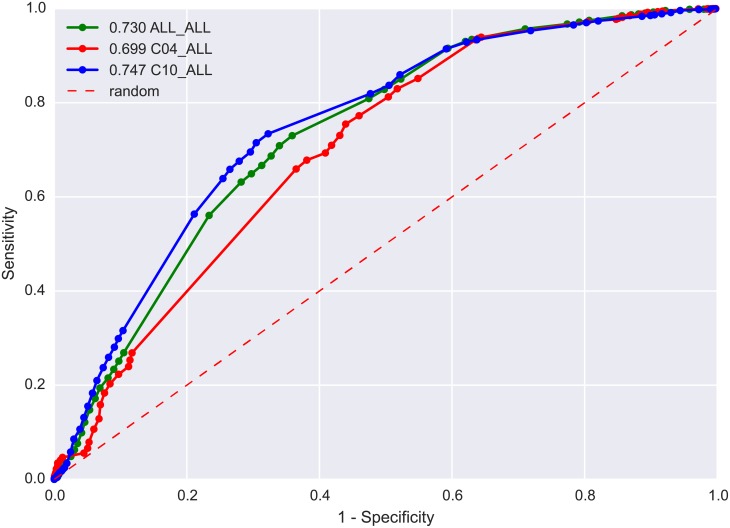
Validating inferred has_indication associations. All 18,889 has_indication associations captured in our integrated network were extracted. These associations were used as a means of validating the ability of our approach to identify known has_indication associations. *Note*: For each disease category (ALL, C04 and C10) the set of known indications were pruned to only include those containing drugs included in the inferences made by our approach (totalling 17,883). Mapping was done using a *Sim* value of 0.633, this is equivalent to a distance of two nodes in the MeSH hierarchy.

We then looked at how well our methodology was able to identify known has_indication associations captured in our network. All has_indication associations (from the four sources listed in Table 2) that involved the 1,188 approved small molecules used during the search were extracted (S4 Table). Fig 5 shows how the approach performs in identifying known has_indication associations for different therapeutic areas (all, C04 and C10) and different disease types (Common_Diseases and Rare_Diseases). Of the 18,889 known has_indication associations, 1,006 involved 63 drugs that were part of the 1,188 investigated, but returned no mappings, leaving 17,883 that could potentially be validated. For mapping known has_indication associations to those inferred by our approach we use a Sim threshold of 0.633, the equivalent of a 2 node distance within the MeSH hierarchy. We believe this provides the best trade-off between the verbosity of the MeSH hierarchy whilst also ensuring inferred diseases are close enough in disease mechanism for the proposed therapeutic small molecule to be relevant. Using the Sim threshold of 0.633, our approach identifies 12,955 of the known has_indication associations (72.65%) (S5 Table). An AUC of 0.73 was achieved when looking at all of the inferred dd associations (Fig 5). The number of knowns identified by the approach can be increased to 97.6% if the Sim value is relaxed to 0.231, which represents a node distance of nine in the MeSH hierarchy (S5 Table).

#### C04: Neoplasms

55,875 unique has_indication inferred associations involved neoplasms (branch C04 of the MeSH hierarchy). 16,492 of these unique associations involve Rare_Diseases (inferred from 29,331 mappings) whilst 39,383 unique has_indication associations involving Common_Diseases were identified (inferred from 73,501 mappings). Of the 2,856 known has_indication dd associations our approach identifies 1,927 of these, or 68%. When we consider the fact that 455 of the knowns involve 28 drugs that our approach was unable to infer associations for, due to lack of data, that gives us an 80% identification rate for known dd associations involving neoplasms, with an AUC of 0.69 (Fig 5).

Of the top 10 ranked inferred dd associations involving neoplasms (Table 4), we see that three map exactly to indications in our network, one is currently being investigated in a clinical trial [65], one has been previously investigated in the clinic [64], one is now approved for the indication we propose and another is supported by literature [66]. Of the top 10 inferred indications, three are novel and are currently not supported by evidence. One of those indications, is the use of Pazopanib in the treatment of Mastocytosis.

**Table 4:** Top 10 inferred associations involving unique neoplasm diseases.

Drug (*DrugBank*)	Gene	Disease (*MeSH UI*)	Type (*ORDO*)	Evidence	Score
Sunitinib (*DB01268*)	*KIT*	Gastrointestinal Stromal Tumors (*D046152*)	R (*44890*)	M(1.0)	0.999
Ponatinib (*DB08901*)	*FLT3*	Acute myeloid leukemia *D015470*	R (*519*)	A	0.998
Dasatinib (*DB01254*)	*EPHB2*	Familial prostate cancer (*C537243*)	R (*1331*)	C [64]	0.996
Ethinyl Estradiol (*DB00977*)	*ESR1*	Breast Neoplasms (*D001943*)	C	M(1.0)	0.988
Dasatinib (*DB01254*)	*BCR*	Myelogenous, Chronic, BCR-ABL Positive (*D015464*)	C	M(1.0)	0.988
Pazopanib (*DB08901*)	*KIT*	Mastocytosis *D008415*	R (*98292*)	-	0.984
Afatinib (*DB08916*)	*ERBB2*	Stomach Neoplasms (*D013274*)	C	-	0.973
Sunitinib (*DB01268*)	*RET*	Multiple endocrine neoplasia type 2B (*D018814*)	R (*247709*)	-	0.961
Sunitinib (*DB01268*)	*RET*	Pheochromocytoma (*D010673*)	C	C [65]	0.960
Sunitinib (*DB01268*)	*NTRK1*	Familial medullary thyroid carcinoma (*C536911*)	R (*99361*)	P [66]	0.958

We present the top ranked 10 inferred has_indication associations involving neoplasms. All ranked associations are available for download. A disease is classed as Rare (*R*) if it maps to ORDO and Common (*C*) if it is only in MeSH and not mappable to an ORDO concept. Evidence: *M* = maps to indications in dataset with *Sim* 0.66 or above; *A* = approved; *C* = clinical trial; and *P* = scientific paper.

#### Pazopanib as a treatment for Mastocytosis?

Pazopanib is a small molecule inhibitor of multiple protein tyrosine kinases and is approved for the treatment of advanced renal cell carcinoma and advanced soft tissue sarcomas. Mastocytosis, classed as a rare disease, is a mast cell activation disorder of both children and adults caused by the presence of too many mast cells (mastocytes) and CD34+ mast cell precursors. The cause of mastocytosis is not known but activating mutations in the proto-oncogene receptor tyrosine kinase, *KIT*, are found in most patients with mastocytosis [67]. The mutation makes mast cells more sensitive to stem cell factor (SCF). SCF plays an important role in stimulating the production and survival of cells such as blood cells and mast cells, inside the bone marrow. When bone marrow is exposed to SCF, it produces more mast cells than the body can cope with, leading to symptoms of mastocytosis [67]. Although no official treatment exists for mastocytosis many drugs are prescribed off-label, including the tyrosine kinase inhibitors, desatinib, imatinib and masitinib [67]. Due to the fact that that pazopanib displays inhibitory effects on the *KIT* enzyme similar to those that have been used as off-label treatments, it poses an interesting alternative in the treatment of mastocytosis.

#### C10: Nervous System Diseases

54,635 unique has_indication inferred associations involved diseases of the nervous system (branch C10 of the MeSH hierarchy). 13,394 of these unique associations involve Rare_Diseases (inferred from 17,677 mappings) whilst 41,241 unique has_indication associations involving Common_Diseases were identified (inferred from 66,536 mappings). Of the 4,249 known has_indication dd associations our approach identifies 2,846 of these. When we consider the fact that 125 of the knowns involve 37 drugs that, due to holes in the data, our approach was unable to infer associations for, that gives us a 69.0% identification rate for known dd associations involving nervous system diseases, with an AUC of 0.75 (Fig 5).

Of the top 10 ranked inferred dd associations involving diseases of the nervous system (Table 5), we see that only one maps exactly to an indication in our network whilst another maps with a Sim of 0.66 (MeSH distance of two nodes). Another eight are novel and are currently not supported by evidence. One of those indications is the use of Lisinopril in the treatment of Alzheimer’s Disease.

**Table 5:** Top 10 inferred associations involving unique diseases of the nervous system.

Drug (*DrugBank ID*)	Gene	Disease (*MeSH UIs*)	Type (*ORDO*)	Evidence	Score
Nitrendipine (*DB01054*)	*CACNA1S*	Hypokalemic periodic paralysis (*D020514*)	R (*681*)	-	0.999
Clonazepam (*DB01068*)	*GABRA1*	Juvenile myoclonic epilepsy (*D020190*)	R (*307*)	M (0.76)	0.999
Mifepristone (*DB00834*)	*ESR1*	Bulbospinal neuronopathy, X-linked recessive (*C537017*)	C	-	0.999
Memantine (*DB01043*)	*GRIN2A*	Landau-Kleffner Syndrome (*D018887*)	R (*98818*)	-	0.996
Bromocriptine (*DB01200*)	*DRD2*	Myoclonus-dystonia syndrome (*C536096*)	R (*36899*)	-	0.994
Roflumilast (*DB01656*)	*PDE4D*	Acrodysostosis (*C538179*)	R (*950*)	-	0.991
Lisinopril (*DB00722*)	*ACE*	Alzheimer Disease (*D000544*)	C	-	0.991
Roflumilast (*DB01656*)	*PDE4D*	Stroke (*D020521*)	C		0.987
Clonazepam (*DB01068*)	*GABRB3*	Epilepsy, Absence (*D004832*)	C	M (1.0)	0.991
Triazolam* (*DB00897*)	*GABRG2*	Generalized Epilepsy With Febrile Seizures Plus, Type 3 (*C565811*)	C	-	0.988

We present the top ranked 10 inferred has_indication associations involving unique diseases of the central nervous system. All ranked associations are available for download. A disease is classed as Rare (*R*) if it maps to ORDO and Common (*C*) if it is only in MeSH and not mappable to an ORDO concept. Evidence: *M* = maps to indications in dataset with *Sim* 0.66 or above; *A* = approved; *C* = clinical trial; and *P* = scientific paper. (*This drug has been withdrawn in the UK due to risk of psychiatric adverse drug reactions, but continues to be available in the U.S)

#### Lisinopril as a treatment for Alzheimer’s Disease?

Alzheimer’s Disease is a chronic neurodegenerative disease that usually starts slowly and gets worse over time, and currently has no cure. Lisinopril, a potent, competitive inhibitor of angiotensin-converting enzyme (ACE), is used to treat hypertension and symptomatic congestive heart failure. There is evidence to suggest that Angiotensin converting enzyme inhibitors can reduce the risk of Alzheimer’s disease in the absence of apolipoprotein E4 allele [68]. As such, we propose lisinopril as a potential treatment for Alzheimer’s disease.

## Discussion

In this paper, we explored the concept of using a data driven approach to infer novel drug repositioning leads; our results identify diseases of the nervous system as being in need of more small molecule treatments. We integrated and ranked G-D associations from multiple datasources, highlighting the need for a standard representation within the field. We use these ranked associations to create a semantically-rich integrated network for drug repositioning. We show how mining this network for semantic subgraphs allows us to infer novel dd interactions.

We identify two therapeutic areas to focus on, one, C04, with a relatively rich knowledge base, and one, C10, containing many diseases that are currently in need of a therapeutic molecule. We see, as expected, that the approach performs better when looking at neoplasms (C04) in comparison to the less treated and less informed diseases of the nervous system (C10); highlighting the fact that systems approaches are limited by the data available. This limit in data may become more of a problem in the long term, especially when it comes to developing treatments for diseases of the central nervous system. Clinical trials are very expensive in the area of nervous system diseases, due to the placebo affect, meaning that great numbers of trialists are needed. As a result many companies are withdrawing their development efforts from this area, making nervous system diseases a great area of opportunity for repositioning, and in particular *in silico* approaches. Our approach does not address the problems caused by the placebo affect. Rather, by bringing data together, in a similar fashion to the clinician, we hope that as more data becomes available, we can reduce the attrition rates whilst also improving efficacy.

The approach presented here makes use of a MeSH distance measure, Sim. This measure is used twice during the approach. A Sim value of 0.768 is used for filtering potential has_side_effect associations, equivalent to a one node path from a known side effect. A lower Sim value of 0.633 is used to validate inferred has_indication associations against the known indications captured in the network, equivalent to a two node path. The two values vary due to the fact that they are used for different purposes. Reducing the stringency used to filter potential side effects, results in the loss of many of the true positives (S3 Table). Indeed, by filtering potential side effects using a Sim of 0.633 instead of 0.768 would result in a loss of 31% of true positive inferred has_indication associations. When validating inferred has_indication associations, the lower the Sim value the greater the AUC (S6 Fig). In this instance, a Sim value of 0.633 gives the best trade off between AUC and maintaining semantic ‘equivalence’, when it comes to validation. We believe that these differing Sim values reflect the manner in which drugs are marketed, with indications being as high level as possible for marketing reasons. On the other hand side effects tend to map to a greater level of granularity and so do not require such lenient mapping.

Possible extensions to this approach should include more thorough analysis in terms of the identification of disease areas of interest. Instead of simply identifying a therapeutic area that appears to be relatively untreated one could consider other factors for disease prioritisation. For example not all diseases have the same impact on society and so integrating data that considers this would be useful. The WHO global burden of disease measures burden of disease using the disability-adjusted-life-year (DALY).

As well as a more thorough disease prioritisation step more focus must be placed on directionality, both in terms of the effect on function of the gene mutation and the drug functionality (e.g. agonist, antagonist). As far as we are aware no datasource details the effect of function that a gene mutation has; i.e. does it result in LoF (the gene product has less or no function) or GoF mutation (product of mutated gene gains a new and abnormal function) and although we used a text mining approach to try and address this it was not exhaustive. Drug functionality must also be considered if this work is truly to provide detailed inferences. We did manage to get drug functionality for around 500 drugs from ChEMBL, but this did not cover all drugs in the dataset. This problem is highlighted with by the first ranked inferred association from the diseases of the central nervous system (Table 5). We propose Nitrendipine, a potent blocker of the calcium channel (CACNA1S), as a treatment for Hypokalemic periodic paralysis. Although both the binds_to and involved_in associations are correct, the lack of directionality attached to the G-D association makes this particular inference a poor one. Nitrendipine is annotated as being an inhibitor of CACNA1S in our dataset, as such if the mutation involved of CACNA1S had been correctly annotated as a LoF mutation, this inference would have been filtered as contradictory. As such, the administration of Nitrendipine as a treatment for Hypokalemic periodic paralysis is likely to exacerbate the condition as opposed to treating it.

Despite this approach allowing for an initial reduction of the search space the next step would require a more robust filtering of the results. One would need to ensure that the target could indeed be reached by the drug, i.e. if a compound is unable to pass the membrane the target must be located on the surface of the cell. This could be achieved by looking at cellular location of targets, which could be extracted from GOA, as well as the physiochemical properties of the compound, from DrugBank or ChEMBL.

We have introduced a strategy for mining for potential drug repositioning opportunities, however, at the moment, we can see how this is limited by the data we have available. We pave the way for more stringent ontological representation of G-D associations; like the Experimental Factor Ontology (EFO) work being carried out at the Centre for Therapeutic Target Validation (CTTV). We believe that as the quality of data increases this *in silico* approach will complement target identification and validation; reducing target attrition through efficacy.

## Supporting Information

S1 Article Gain of Function & Loss of Function Gene-Disease Associations. Article describes the rational and methods used during the extraction of the gain-of-function & loss-of-function gene-disease associations.
(PDF)

S1 Fig Metagraph of the integrated dataset. Metagraph shows the node types and the edge types used in the integrated dataset and how they interact with one another.
(EPS)

S2 Fig Identifying an area of unmet need. Using Eq 3 we scored each therapeutic area in the MeSH hierarchy. The TAU score considers how mush data is captured in our dataset and the percentage of diseases in that therapeutic area that do not have a marketed small therapeutic molecule.
(EPS)

S3 Fig Identifying a therapeutic area to validate our approach. Using Eq 4 we score each therapeutic area in the MeSH hierarchy. The RTA score considers how much data is captured in our dataset for each therapeutic area. *Note*: red diamonds show the therapeutic areas which include <3.44% (100/29) of all diseases. For the purpose of this exercise they will not be considered for analysis as they do not offer a fair representation of the data included in the work.
(EPS)

S4 Fig ROC curve when altering D-value used to score associations with UniProt as the gold standard. Using UniProt as the gold standard, all G-D associations were scored using D-values from 1.0–8.0. We see that a D-value (DV) of 5.0 (grey) gives us the highest area under the curve AUC when validating using UniProt.
(EPS)

S5 Fig Calculating *Sim* threshold for pruning potential side effects from inferred indications. This figure provides a graphical representation of the data captured in S3 Table. For each threshold the F-Measure (using precision and recall of known indications captured in the network), shown in black, as well as the average ranking position of the excluded potential side effects, shown in red, were calculated. In order to calculate the ranking positions of those excluded, all associations inferred by the methodology were ranked prior to any filtering and it was these rankings used here. The aim of filtering out potential side effects was to reduce noise in the results whilst also ensuring we weren’t filtering potential indications. We assume that the associations scoring higher, and thus rank higher (highest being 1), are predicted with more confidence and thus we wish to ensure potential side effects are excluded from the highest ranking associations.
(EPS)

S6 Fig Altering *Sim* threshold whilst mapping inferred has_indication associations to our set of 18,889 known. For each of the *Sim* values investigated we see how many of the known has_indication associations are identified by the inferred has_indication associations. Unsurprisingly, as the *Sim* value is relaxed the AUC increases.
(EPS)

S1 Table Number of mappings returned for each MeSH therapeutic area after filtering. Associations that include diseases that fall under multiple MeSH categories are duplicated in the counts (if a disease has multiple mesh tree terms from the same therapeutic area these are also counted multiple times). Only associations that survived the filtering steps are included.
(PDF)

S2 Table LLS score for each test source when altering gold standard (GS) source. After applying the LLS method and alternating the gold standard, GS sources (left column), we see how every other source, the test sources (top row) perform in terms of identifying the ‘knowns’ captured in the GS. Performance is measured using the LLS score, which is shown. Furthermore, for each GS used, test sources are ranked in terms of performance (the higher the LLS score the better the performance of that test source). All ranks are shown in brackets and all scores are rounded to 2 decimal places.
(PDF)

S3 Table*F*_1_ score using each of the possible *Sim* scores whilst pruning potential side effects from all mappings returned during the search.Predicted interactions were mapped to the known indications using a *Sim* of 1.0. *Note*: all values are corrected to 4 d.p. $F_{1}=\left(2\times \frac{P\times R}{P+R}\right)$ TP = true positive, FP = false positive, FN = false negative.(PDF)

S4 Table Number of has_indication associations captured in the network that involve the 1,188 approved small molecules. Sources cumulatively provide 18,889 unique has_indication associations which is reduced to 17,883 when only considering those involving drugs captured in the inferences made by our approach. Percentage in brackets reflects the percentage of associations from source *x* that involves drugs found in both sets (source *x* and the inferences).
(PDF)

S5 Table Number of known has_indication associations mapped to inferred associations using altering *Sim* values. Of the 18,889, known has_indication associations, 1,006 involved 63 drugs of the 1,188 investigated for which our approach returned no mappings, leaving 17,883 that could potentially be validated.
(PDF)

S1 Data Mapping of GWAS traits to MeSH headers used during the work. (TXT)

S2 Data A TSV version of the integrated dataset used. (ZIP)

S3 Data All 309,885 scored and ranked gene-disease associations. (TXT)

S4 Data All 275,934 scored and ranked inferred drug-disease associations. (TXT)
