# Supplementary material for: An Integrated Data Driven Approach to Drug Repositioning Using Gene-Disease Associations
Source: PLoS One. 2016 May 19;11(5):e0155811. doi: 10.1371/journal.pone.0155811 (PMC4873016; doi:10.1371/journal.pone.0155811)
Supplement: S1 Article — Article describes the rational and methods used during the extraction of the gain-of-function & loss-of-function gene-disease associations. (PDF) [file pone.0155811.s001.pdf]

Article describes the rational and methods used during the extraction of the gain-of-function & loss-of-function gene-disease associations.

One limitation with the various methods of data collection is the lack of associations annotated with the gene functionality associated to the disease (i.e. loss-of-function (LoF) or gain-of-function (GoF)). The GoF and LoF searches were performed across Medline abstracts using automatic text mining with Linguamatics I2E. Sentences needed to contain three semantic entities: a disease, a gene and a loss or gain of function phrase. For the gain of function any of the following phrases were sought:

1. gain-of-function
2. gain of function
3. activating mutation(and morphological variants of activating);

similar was sought for the loss of function. To increase accuracy, disease and gene semantic terms were filtered to exclude the most ambiguous terms (by using Linguamatics I2Es disambiguation score ≤ 75). The disease terms were automatically mapped to MeSH and the genes to NCBI Entrez Gene Id. The data was prepared as a triplet: disease, gene, GoF/LoF. These associations were produced on 16th October 2015. It was then necessary to map between the MeSH tree terms and the MeSH UIs with stats shown below in Table 1.

Table 1: **GoF and LoF gene-disease associations.**

**Type**	**#Associations**	**#Unique**	**#Mappable to MeSH**
GoF	16.3k	1,734	1,248
LoF	29k	3,059	2,211

Breakdown of the GoF and LoF gene-disease associations captured using linguamatics and the number of these successfully mapped to MeSH UIs.
