# Supplementary material for: An Integrated Data Driven Approach to Drug Repositioning Using Gene-Disease Associations
Source: PLoS One. 2016 May 19;11(5):e0155811. doi: 10.1371/journal.pone.0155811 (PMC4873016; doi:10.1371/journal.pone.0155811)
Supplement: S1 Table — Associations that include diseases that fall under multiple MeSH categories are duplicated in the counts (if a disease has multiple mesh tree terms from the same therapeutic area these are also counted multiple times). Only associations that survived the filtering steps are included. (PDF) [file pone.0155811.s008.pdf]

**Therapeutic Area**	**# of mappings**
**[C01]** bacterial infections and mycoses	7,477
**[C02]** virus diseases	8,359
**[C03]** parasitic diseases	2,284
**[C04]** neoplasms	55,875
**[C05]** musculoskeletal diseases	14,059
**[C06]** digestive system diseases	21,427
**[C07]** stomatognathic diseases	5,617
**[C08]** respiratory tract diseases	13,519
**[C09]** otorhinolaryngologic diseases	2,409
**[C10]** nervous system diseases	54,635
**[C11]** eye diseases	9,773
**[C12]** urologic and male genital diseases	13,681
**[C13]** female genital diseases and pregnancy complications	18,097
**[C14]** cardiovascular diseases	26,313
**[C15]** hemic and lymphatic diseases	15,160
**[C16]** congenital, hereditary, and neonatal diseases and abnormalities	32,089
**[C17]** skin and connective tissue diseases	13,975
**[C18]** nutritional and metabolic diseases	18,628
**[C19]** endocrine system diseases	16,667
**[C20]** immune system diseases	16,943
**[C21]** disorders of environmental origin	2
**[C22]** animal diseases	757
**[C23]** pathological conditions, signs and symptoms	38,232
**[C24]** occupational diseases	347
**[C25]** chemically-induced disorders	5,372
**[C26]** wounds and injuries	1,933
**[F01]** behavior and behavior mechanisms	5,196
**[F02]** psychological phenomena and processes	1,101
**[F03]** mental disorders	23,406
