# Supplementary material for: An Integrated Data Driven Approach to Drug Repositioning Using Gene-Disease Associations
Source: PLoS One. 2016 May 19;11(5):e0155811. doi: 10.1371/journal.pone.0155811 (PMC4873016; doi:10.1371/journal.pone.0155811)
Supplement: S2 Table — After applying the LLS method and alternating the gold standard, GS sources (left column), we see how every other source, the test sources (top row) perform in terms of identifying the ‘knowns’ captured in the GS. Performance is measured using the LLS score, which is shown. Furthermore, for each GS used, test sources are ranked in terms of performance (the higher the LLS score the better the performance of that test source). All ranks are shown in brackets and all scores are rounded to 2 decimal places. (PDF) [file pone.0155811.s009.pdf]

	Test Sources									
GS Sources	**BEF**	**CTD**	**GOF**	**GWA**	**MGD**	**OMI**	**ORP**	**RGD**	**SEM**	**UNI**
**BEFREE**	-	9.26 (6)	9.97(5)	8.05 (9)	10.32 (4)	11.21 (2)	10.68 (3)	8.34 (8)	8.55 (7)	**11.21 (1)**
**CTD**	8.76 (9)	-	9.49 (7)	9.8 (5)	12.32 (3)	**14.12 (1)**	12.21 (4)	9.52 (6)	8.85 (8)	13.82 (2)
**GOFLOF**	10.91 (7)	11.38 (6)	-	10.11 (9)	12.92 (4)	13.59 (3)	13.83 (2)	10.61 (8)	11.46 (5)	**14.53 (1)**
**GWAS**	9.62 (7)	10.86 (4)	8.92 (9)	-	11.24 (3)	**12.06 (1)**	10.81 (5)	9.19 (8)	10.1 (6)	12.0 (2)
**MGD**	11.81 (8)	14.16 (4)	13.09 (5)	11.77 (9)	-	15.79 (2)	15.04 (3)	12.29 (7)	12.37 (6)	**15.91 (1)**
**OMIM**	11.06 (9)	14.01 (4)	12.35 (5)	11.44 (6)	14.9 (2)	-	14.6 (3)	11.15 (8)	11.43 (7)	**16.78 (1)**
**ORPHANET**	12.65 (9)	15.59 (4)	13.29 (7)	12.77 (8)	16.22 (3)	17.4 (2)	-	13.66 (6)	13.72 (5)	**17.5 (1)**
**RGD**	9.41 (9)	10.52 (5)	10.06 (6)	9.52 (8)	11.5 (3)	11.29 (4)	**11.73 (1)**	-	9.83 (7)	11.71 (2)
**SEMREP**	9.09 (8)	9.78 (6)	10.73 (5)	9.05 (9)	11.03 (3)	11.38 (2)	11.02 (4)	9.27 (7)	-	**11.5 (1)**
**UNIPROT**	11.19 (8)	14.08 (4)	12.4 (5)	10.95 (9)	14.97 (2)	**16.57 (1)**	14.68 (3)	11.26 (7)	11.59 (6)	-
Mean	8.22 (10)	4.77 (5)	6 (6)	8 (9)	3 (3)	2.11 (2)	3.11 (4)	7.22 (8)	6.33 (7)	**1.33 (1)**
