# Supplementary material for: An Integrated Data Driven Approach to Drug Repositioning Using Gene-Disease Associations
Source: PLoS One. 2016 May 19;11(5):e0155811. doi: 10.1371/journal.pone.0155811 (PMC4873016; doi:10.1371/journal.pone.0155811)
Supplement: S3 Table — Predicted interactions were mapped to the known indications using a Sim of 1.0. Note: all values are corrected to 4 d.p. F1=(2×P×RP+R) TP = true positive, FP = false positive, FN = false negative. (PDF) [file pone.0155811.s010.pdf]

Sim	# of indications	TP	FP	FN	Precision (P)	Recall (R)	F-measure (F_1)	Mean Rank of Pruned SEs
1	295742	7494	288248	0	0.0253	1	0.0494	155528
0.7686	275934	6052	269882	1442	0.0219	0.8076	0.0427	133923
0.6333	239649	4204	235445	3290	0.0175	0.561	0.034	137806
0.5372	206227	3181	203046	4313	0.0154	0.4245	0.0298	141460
0.4628	180834	2618	178216	4876	0.0145	0.3493	0.0278	143843
0.4019	164765	2370	162395	5124	0.0144	0.3163	0.0275	145038
0.3504	156170	2248	153922	5246	0.0144	0.3	0.0275	145812
0.3059	152523	2209	150314	5285	0.0145	0.2948	0.0276	146241
0.2665	151287	2193	149094	5301	0.0145	0.2926	0.0276	146410
0.2314	150929	2188	148741	5306	0.0145	0.292	0.0276	146454
0.1996	150857	2188	148669	5306	0.0145	0.292	0.0276	146470
