# Supplementary material for: An Integrated Data Driven Approach to Drug Repositioning Using Gene-Disease Associations
Source: PLoS One. 2016 May 19;11(5):e0155811. doi: 10.1371/journal.pone.0155811 (PMC4873016; doi:10.1371/journal.pone.0155811)
Supplement: S4 Table — Sources cumulatively provide 18,889 unique has_indication associations which is reduced to 17,883 when only considering those involving drugs captured in the inferences made by our approach. Percentage in brackets reflects the percentage of associations from source x that involves drugs found in both sets (source x and the inferences). (PDF) [file pone.0155811.s011.pdf]

**Source**	**# has_indication (% of total from source)**
CTD	14,761 (79.6)
SIDER4	3,641 (81.1)
PREDICT	1,210 (95.7)
NDFRT	2,586 (58.8)
