# Supplementary material for: An Integrated Data Driven Approach to Drug Repositioning Using Gene-Disease Associations
Source: PLoS One. 2016 May 19;11(5):e0155811. doi: 10.1371/journal.pone.0155811 (PMC4873016; doi:10.1371/journal.pone.0155811)
Supplement: S5 Table — Of the 18,889, known has_indication associations, 1,006 involved 63 drugs of the 1,188 investigated for which our approach returned no mappings, leaving 17,883 that could potentially be validated. (PDF) [file pone.0155811.s012.pdf]

Sim	**# known has_indication (% of total)**
1.0	6,114 (34.2)
0.768	9,188 (51.38)
0.633	12,955 (72.65)
0.537	15,527 (87.10)
0.462	16,689 (93.60)
0.401	17,122 (95.74)
0.350	17,321 (96.88)
0.305	17,388 (97.50)
0.266	17,401 (97.58)
0.2314	17,407(97.6)
